# Neuro-meningeal cryptococcal infection revealing a multiple myeloma

**DOI:** 10.11604/pamj.2020.36.324.20407

**Published:** 2020-08-21

**Authors:** Sameh Sayhi, Sawsen Bouzidi, Imen Beji, Aman Allah Nasr, Souha Hannachi, Ines Bedoui, Samy Layouni, Najiba Fekih-Mrissa, Bassem Louzir, Brahim Nsiri, Rym Abid, Riadh Battikh

**Affiliations:** 1 Department of Internal Medicine, Military Hospital, Tunis, Tunisia,; 2 Laboratory of Hematology, Military Hospital, Tunis, Tunisia,; 3 Department of Neurology, Military Hospital, Tunis, Tunisia,; 4 Molecular Biology Unit (UR17DN06), Laboratory of Hematology, Military Hospital, Tunis, Tunisia

**Keywords:** Cryptococcal-meningitis, multiple myeloma, hypogammaglobulinemia, lymphopenia

## Abstract

Rare cases of Cryptococcus have been documented in patients living with multiple myeloma. To date there has been no documented evidence of cryptococcosis revealing multiple myeloma. We reported a 63-year-old man who had a 2-months history continuous holocranial headaches, morning vomiting, complaining of blurred vision and fever. The biologic and the imaging showed a Cryptococcus meningoencephalitis. The search for a cause of immunodeficiency revealed a multiple myeloma. The diagnosis for Cryptococcus was confirmed according to an India ink stain, blood and cerebrospinal fluid culture. The patient's treatment for multiple myeloma was initiated with a chemotherapy regimen. The evolution was good without complication. Cryptococcosis, especially in the neuro-meningeal form, is a serious, deadly opportunistic infection. The search of an underlining immunodeficiency must be systematic. In this case, it was associated with early stage multiple myeloma.

## Introduction

Cryptococcosis is an opportunistic fungal infection that predominantly affects immuno-compromised hosts [1]. Often it is an opportunistic infection in immuno-compromised patients such as those with Human Immunodeficiency Virus (HIV), those undergoing glucocorticoid treatments (e.g. post organ transplantation), and oncological treatments [2]. The most frequent presentation of cryptococcal infection is subacute meningitis. Rare cases of Cryptococcus have been documented in patients living with multiple myeloma (MM) [2,3]; however, to date there has been no documented evidence of cryptococcosis revealing multiple myeloma. This research was approved by the local ethics committee and informed written consent was obtained from the patient for publication of this case report and accompanying image.

## Patient and observation

A 63-year-old man presented with persistent, gradually aggravating headaches over a period of two months. They were continuous holocranial headaches evolving in a context of impaired general performance status. Subsequently, the patient presented again three weeks later with morning vomiting and complaining of blurred vision. The patient reported having been febrile for the preceding two months. His past medical history was unremarkable and he was not currently on medication. He was then admitted to the hospital of Tunis in September 2018 for an exploration of chronic febrile illness and headaches. The physical examination recorded a temperature of 38.5°C. The neurological exam showed a conscious patient well oriented in space and time who had neither meningeal nor vestibular syndrome. A bilateral paralysis of the sixth cranial nerve was found associated with bilateral hypo-acousia. However, he had no sensori- motor deficit and no neurological localizing signs. An eye fundus revealed a bilateral stage I papilledema. All others clinical examinations were normal. A basic metabolic panel revealed several abnormalities: an elevated C-reactive protein, lymphopenia, hyper-fibrinogenemia, and anemia (Table 1). A cerebral computed tomography scan and magnetic resonance imaging showed normal findings. However, an audiogram showed bilateral perceptive deafness. A lumbar puncture revealed an opening cerebrospinal fluid (CSF) pressure of 350mm of water, protein 0.69 g/L, glucose 0.3mmol/L, and WBC of 60x10^6^/L (80% lymphocytes). An India ink stain was positive for Cryptococcus. CSF and serum cryptococcal antigen tests also returned positive (with titers of 1:10000 and 1:1000 respectively). Blood and CSF culture were positive and revealed the presence of *Cryptococcus neoformans*. The antifungal sensitivity test was also performed (Table 1). Therefore, a diagnosis of Cryptococcus meningoencephalitis was established.

**Table 1:** laboratory data on admission

Laboratory Test	Patient Value	Normal Range
	**Biochemistry**	
**Blood urea (H)**	**4.8 mmol/L**	**3.3-7 mmol/L**
**C-reactive protein (H)**	**34 mg/L**	**< 5 mg/L**
**Sodium (L)**	**134 mmol/L**	**136-145 mmol/L**
**Chloride (L)**	**97 mmol/L**	**101-111 mmol/L**
**Protein (L)**	**63 g/L**	**65-80 g/L**
Potassium	3.5 mmol/L	3.5-4.5 mmol/L
Bicarbonate	27 mmol/L	22-28 mmol/L
Calcemia	2.27 mmol/L	2.25-2.60 mmol/L
Phosphoremia	1.2 mmol/L	0.80-1.61 mmol/L
Plasma creatinine	60 μmol/L	60-120 μmol/L
Total bilirubin	9 μmol/L	<17 μmol/L
PAL	83 UI/L	42-121 UI/L
AST	13 UI/L	10-42 UI/L
ALT	18 UI/L	10-60 UI/L
LDH	108 UI/lL	91-260 UI/L
	**Hematology**	
**Lymphocytes (L)**	**0.85x10^9^ /L (13.2 %)**	**1.5-4x10^9^ /L**
**Hemoglobin (L)**	**11 g/dL**	**13-17 g/dL**
White blood cells	6.5x10^9^/L	4-10x10^9^ /L
Neutrophils	5x10^9^ /L (77.4 %)	1.7-7 x10^9^ /L
Monocytes	0.54x10^9^ /L (8.4%)	0.1-1 x10^9^ /L
Basophils	0x10^9^ /L (0%)	<0.1x10^9^ /L
Eosinophils	0.06x10^9^ /L (1%)	<0.5x10^9^/L
Mean corpuscular volume	86 fl	80-100 fl
Platelet	154x10^9^ /L	150-450x10^9^ /L
	**Coagulation**	
Prothrombin time activity	70%	70-100%
APTT	32 sec	32 sec
Fibrinogen	4.37 g/L	2-4 g/L
	**Cerebrospinal fluid**	
**WBC (H)**	**60x10^6^ /L**	**< 5x10^6^ /L**
**Protein (H)**	**0.69 g/L**	**< 0.3 g/L**
**Glucose (L)**	**0.3 mmol/L**	**2.5-4.4 mmol/L**
Lymphocytes	80%	-
Neutrophils	20%	-
	**Serum free light chains**	
**lambda (λ) (H)**	**183.14 mg/L**	**5.71-26.30 mg/L**
kappa (κ)	9.47 mg/L	3.30-19.40 mg/L
**Ratio κ / λ (L)**	**<0.10**	**0.26-1.65**


Bold indicates out-of-range. (**H**:high, **L**:low)

**Table 2:** antifungal sensitivity testing of *Cryptococcus neoformans* by Vitek®2 and E-test

Antifungal	MIC (μg/ml)	Vitek®2
AmphotericinB	0.064	Sensitive
Fluconazole	0.5	Sensitive
Voriconazole	0.47	-
Caspofungine	>32	-
Flucytosine	-	Sensitive

**Figure 1:**
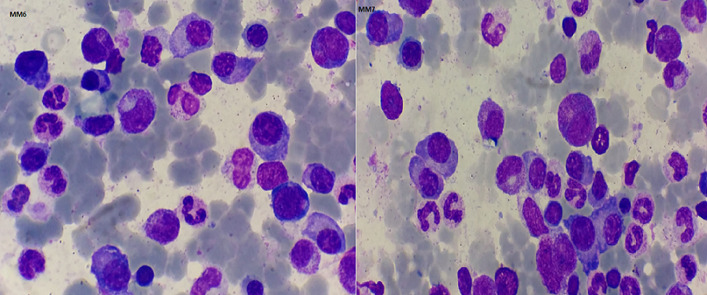
bone marrow aspirate showing myeloma cells (May-Grünwald-Giemsa-stain x1000)

The search for a cause of immunodeficiency that would explain this opportunistic infection, in a seemingly immuno-competent patient, was initiated. The serological testing for hepatitis B virus (HBV), hepatitis C virus (HCV), and human immunodeficiency virus (HIV) were negative (CD4/CD8 ratio was 1.5). Serum protein electrophoresis revealed hypo-albuminemia 23.7 g/l (normal range: 37.7-45.6 g/l) with hypogammaglobulinemia 4.5g/l (normal range: 6.4-12.7g/l). Immunofixation electrophoresis detected monoclonal IgA-lambda (IgA-λ) paraproteinemia at 0.6 g/l. The urine immunofixation revealed the presence of Bence Jones proteinuria type light chain lambda at 0.4 g/l. The serum level of IgA, IgG, and IgM concentrations were measured at 4.04 g/l (normal range: 0.80-3.1), 5.4 (normal range 6.5-15), and 0.45 g/l (normal range: 0.55-3.0) respectively. Quantitative evaluation of the free light chains showed excessively high lambda (λ) chains at 183.14 mg/L (normal range: 5.71-26.3 mg/L) and kappa (κ) chains at 9.47 mg/L (normal range: 3.3-19.4 mg/L) with a κ/λ free light chain ratio of <0.1 (normal range: 0.26-1.65). May-Grunwald-Giemsa stained bone marrow aspirate smears revealed an infiltration of 12% malignant plasma cells with atypically formed and partly bi- or multi-nucleated cells (Figure 1). The β2-microglobulin was elevated at 3.82 mg/L (normal range: 0.8-3 mg/L). Based on these findings, consistent with the International Myeloma Working Group diagnostic criteria, a diagnosis of IgA-λ multiple myeloma was determined. The patient was treated for the Cryptococcal infection with amphotericin B at 0.7 mg/kg/day concurrently with flucytosine at 100 mg/kg/day. Repeated CSF drainage was established to reduce CSF pressure. The induction treatment lasted 21 days, after which the CSF fungi culture became negative and the cryptococcal antigen test in serum and CSF both showed a decrease in antigen titers to 1: 10. He was then transitioned to oral fluconazole at 800 mg/day for 12 weeks. Upon the second week of consolidation treatment for the infection, the patient´s treatment for IgA lambda type multiple myeloma was initiated with a chemotherapy regimen based on thalidomide, melphalan, and prednisone. The evolution was good without complication. No clinical or imaging examination evidence of neurologic complications was found during nine months after this episode.

## Discussion

Human cryptococcal infection, usually the result of *C. neoformans*, has been very well documented in patients with cellular immunodeficiency such as those affected with HIV but can also occur under immunosuppressive conditions such as long-term treatment with corticosteroids, transplant patients, and oncological patients (predominantly hematological, e.g. lympho-proliferative syndromes particularly Hodgkin´s lymphoma and chronic lymphocytic leukemia) [2]. Cryptococcal infections have been rarely reported with multiple myeloma in the absence of any precipitating treatment (principally because cell-mediated immunity is often relatively intact) even though humoral immunity is significantly deficient. The underlying factors in a weakened immune response in MM are, as yet, not fully understood. The rare cases of cryptococcal infection in concert with MM were found almost exclusively in patients with advanced stage multiple myeloma that had been treated with chemotherapy and corticosteroid therapy [4,5], or have had autologous stem cell transplantations [3]. Our case is distinctive given that neuro-meningeal cryptococcosis, an infection generally associated with immunodeficiency factors, is reported in a patient without a significant medical history prior to this infection episode and facilitated the discovery of a hematologic malignancy of multiple myeloma.

A likely conjecture supposes that our patient was exposed to the fungus through the pigeons he raises in his garden. The organism usually enters through the lungs, spreads via the blood stream, and seeds within the central nervous system causing meningoencephalitis [6,7]. The etiologic diagnosis of IgA type myeloma was based upon an elevated serum level of IgA together with hypogammaglobulinemia associated with decreased serum levels of non-myeloma immunoglobulins. Multiple myeloma induces humoral immunity deficiency, characterized by hypogammaglobulinemia that is often profoundly associated with disease progression [8,9]. A low CD4+cell count was observed in our patient. In accordance with previous studies, in MM, CD4+ lymphocytes counts fall with progression of the disease and successive therapies, and have been shown to be linked to a poor prognosis and a risk factor for cryptococcosis infection [10]. In patients with MM, cell-mediated immunity is suppressed with primary disease and treatments, such as steroids, proteasome inhibitors, and other immunosuppressive drugs [2]. Our patient exhibited a decreased CD4+/CD8+ ratio.

Several studies have sought to evaluate defects in T-cell frequencies and function in myeloma. At present, there is limited evidence for both decreased antigen-specific T-cell responses and less so for T-dependent B-cell antigen-specific responses. Significant aberrations in T-cell count and function have been described in MM. However, other studies also have described significantly reduced CD4+/CD8+ ratios [11,12]. Patients with multiple myeloma display multiple cellular and humoral immuno-deficiencies, which increase with conventional-dose regimens and high-dose chemotherapy, and constitute an important predisposing factor for opportunistic infections such as Cryptococcus [13]. Cryptococcal meningoencephalitis is a serious life-threatening infection, with high rates of death despite therapy [14]. This diagnosis needs to be considered in every patient with non-specific lingering neurological presentation even when outside of any previously known immunodeficiency factor(s). The clinical presentation of cryptococcosis was not specific. The diagnosis of neuro-meningeal cryptococcosis infection emerged based primarily upon the direct India ink stained mycological examination, identification through culturing, and the antigenic tests [1,15].

## Conclusion

In summary, the immune function in MM incrementally deteriorates as disease progresses to the symptomatic phase. The immunological synapse and cross-talk between numerical imbalances in B-cell, T-cell populations, and in impaired lymphocyte functionality players is not fully elucidated yet in MM, and remains a key area for further investigation. Cryptococcosis, especially in the neuro-meningeal form, is a serious, deadly opportunistic infection. The search of an underlining immunodeficiency must be systematic. In this case, it was associated with early stage multiple myeloma.
